# Transfer to hospital in planned home births: a systematic review

**DOI:** 10.1186/1471-2393-14-179

**Published:** 2014-05-29

**Authors:** Ellen Blix, Merethe Kumle, Hanne Kjærgaard, Pål Øian, Helena E Lindgren

**Affiliations:** 1 Department of Clinical Medicine, Women’s Health and Perinatology Research Group, Faculty of Health Sciences, University of Tromsø, Tromsø, Norway; 2 Department of Clinical Research, University Hospital of North Norway, Tromsø, Norway; 3 Department of Surgery, University Hospital of North Norway, Narvik, Norway; 4 The Research Unit, Women’s and Children’s Health, The Juliane Marie Centre for Women, Children and Reproduction, Copenhagen University Hospital Rigshospitalet, Copenhagen, Denmark; 5 The Institute of Clinical Medicine, Faculty of Health and Medical Sciences, University of Copenhagen, Copenhagen, Denmark; 6 Department of Obstetrics and Gynaecology, University Hospital of North Norway, Tromsø, Norway; 7 Department of Health and Caring Sciences, University of Gothenburg, Gothenburg, Sweden; 8 Department of Women’s and Children’s Health, Karolinska Institutet, Stockholm, Sweden

**Keywords:** Planned home birth, Transfer to hospital, Emergency transfer, Systematic review

## Abstract

**Background:**

There is concern about the safety of homebirths, especially in women transferred to hospital during or after labour. The scope of transfer in planned home births has not been assessed in a systematic review. This review aimed to describe the proportions and indications for transfer from home to hospital during or after labour in planned home births.

**Methods:**

The databases Pubmed, Embase, Cinahl, Svemed+, and the Cochrane Library were searched using the MeSH term “home childbirth”. Inclusion criteria were as follows: the study population was women who chose planned home birth at the onset of labour; the studies were from Western countries; the birth attendant was an authorised midwife or medical doctor; the studies were published in 1985 or later, with data not older than from 1980; and data on transfer from home to hospital were described. Of the 3366 titles identified, 83 full text articles were screened, and 15 met the inclusion criteria. Two of the authors independently extracted the data. Because of the heterogeneity and lack of robustness across the studies, there were considerable risks for bias if performing meta-analyses. A descriptive presentation of the findings was chosen.

**Results:**

Fifteen studies were eligible for inclusion, containing data from 215,257 women. The total proportion of transfer from home to hospital varied from 9.9% to 31.9% across the studies. The most common indication for transfer was labour dystocia, occurring in 5.1% to 9.8% of all women planning for home births. Transfer for indication for foetal distress varied from 1.0% to 3.6%, postpartum haemorrhage from 0% to 0.2% and respiratory problems in the infant from 0.3% to 1.4%. The proportion of emergency transfers varied from 0% to 5.4%.

**Conclusion:**

Future studies should report indications for transfer from home to hospital and provide clear definitions of emergency transfers.

## Background

In Western countries, women planning to give birth at home are transferred to hospital in case of complications, or if conditions indicating a higher risk for adverse outcomes occur. Although a growing body of evidence points to less intervention in labour in low-risk women who planned home births [1-4], there is still a concern about safety. Guidelines on home births state that only low-risk women should be accepted for, or have recommended, home birth [5-8]. “Low-risk women” are defined as women without medical diseases or conditions that may influence outcomes of pregnancy, without serious complications in previous pregnancies, with a single foetus in the cephalic position, and with a spontaneous onset of labour at term [5-8]. Low-risk women are expected to have a low risk for adverse outcomes, such as perinatal death and other serious complications. This does not rule out the possibility that women who are assessed as low-risk upon onset of labour may need interventions or other medical assistance during labour, or immediately after birth.

To the best of our knowledge, the scope of transfer in planned home births has not been assessed in a systematic review. There is little systematic knowledge on the frequency of women and neonates who are transferred from home to hospital in planned home births, and indications for transfers. A systematic review will be useful for women in making informed choices, and for the planning of care for these women.

The aims of the present systematic review were as follows: (1) to describe how often women and neonates are transferred from home to hospital during labour or after birth; (2) to describe the proportion of women transferred for reasons that may indicate higher risks for adverse outcomes, such as “foetal distress”, “postpartum haemorrhage” and “respiratory problems in the infant”; and (3) to describe the proportion and definitions for emergency transfers.

## Methods

A systematic review is a research method that aims to identify and compare individual studies on one topic and summarise their findings. The “MOOSE statement”, which is the recommended guidelines for publication of systematic reviews of observational studies in epidemiology [9], was used to prepare this manuscript. We also used the “PRISMA statement”, which recommends preferred reporting items for systematic reviews and meta-analyses [10].

### Sources

We conducted electronic searches in Medline, Embase, Cinahl, Swemed, and the Cochrane Library combining the MeSH term “home childbirth” to identify all published studies on home births. The reason for using such broad search terms was that all attempts of narrowing the searches led to few citations found. The searches were conducted between September 15^th^ and October 10^th^, 2012, with an update on December 11^th^, 2013. We also searched the reference lists of all relevant studies. Language restrictions were not applied.

### Study selection

Criteria for selecting studies were as follows. For the required population, pregnant women attempted home birth, meaning that they were accepted for a planned home birth at the onset of labour. The included studies had to report at least one of the following outcome measures: proportion (n/N) transferred from home to hospital during labour; n/N transferred from home to hospital after birth”; n/N transferred for the indications of foetal distress, postpartum haemorrhage, and respiratory problems in the neonate; n/N transferred for other reasons; “n/N had emergency transfer during labour”; “n/N had emergency transfer after the birth”; and the definition of emergency transfer in the study.

Studies included were from Western countries, published in 1985 or later, with data not older than 1980. Western countries were defined as North America, Australia, New Zealand, and all countries in Europe except for the previous Soviet Union. The review was limited to include studies from Western countries to achieve some homogeneity across study populations and health care systems. Since the late 1970s, women with an increased risk for adverse outcomes have not been recommended, and usually not accepted, for home birth or birth in other midwifery-led settings. Only studies with births assisted by an authorised midwife or medical doctor were included.

One of the reviewers (EB) conducted the electronic searches, and screened titles and abstracts to remove duplicates and studies that were obviously not relevant. Each study retrieved in full text was independently assessed by two reviewers for quality (EB, MK, or HL). Any disagreement was resolved by conference or by a third reviewer (HK or PØ). Studies including women with booked home births (e.g., women had booked a home birth, but could have been transferred to hospital care during pregnancy), and those with unplanned home births or with “freebirths” (e.g., home births were planned without the assistance of a midwife or a physician) were not included.

Methodological quality was assessed by using the Norwegian Knowledge Centre for the Health Services tool for assessing the risk of bias [11]. Studies were evaluated according to whether they had a prospective design, if analyses were stratified for nulli- and multiparity, if the study population represented at least 75% of the total home birth group, and if information on parity, caregivers, and duration of observational time was described. Studies were scored as either “good” if they met all of the quality criteria, “medium” if they did not meet all of the criteria, but had no serious flaws, and “poor” if they met none of the criteria, or if 50% or more of the study population failed to be included or followed up. Studies scored as poor were excluded from the review.

### Data extraction and analyses

A data extraction form (Additional file 1) was developed according to our study protocol. The data were extracted from each study and entered into the form independently by two reviewers (EB, MK, or HL). Heterogeneity was assessed by calculating inconsistency (*I*^2^), and by visual inspection of data and forest plots [12,13]. Sensitivity analyses were performed to assess the robustness [14]. We assessed whether performing a meta-analyses was appropriate. StatDirect (Version 2.7.9; Cheshire, UK) was used for analyses.

## Results

### Literature searches and study selection

**Figure 1:**
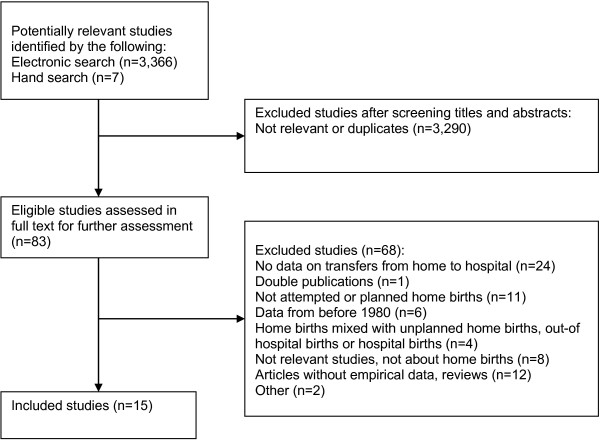
Selection process of eligible studies from all identified studies.

The electronic searches generated 3366 citations. After screening titles and abstracts, 76 studies were retrieved in full text. Searching the literature lists in these 76 articles generated another seven citations. Therefore, 83 studies were reviewed in full text, 15 were included in the review, and 68 were excluded (Figure 1).

There were only minor disagreements in assessing study quality and whether studies should be included. Disagreements were results of oversights and were solved by consensus.

Reasons for exclusions and a bibliography of excluded studies are shown in Additional file 2.

### Description of included studies

Of the 15 included studies, three were from Australia [15-17], three were from Canada [18-20], two were from the USA [21,22], one was jointly from Canada and the USA [4], two were from the UK [1,23], one was from the Netherlands [24], one was from Norway [25], one was from Sweden [26], and one was from Denmark [27]. One study was published in Danish [27], and the others were in English.

The 15 studies included a total of 215,257 women with a planned home birth upon onset of labour. The Dutch study [24] included 168,618 women representing 78% of all women included in the review. The other 14 studies included 46,639 women, and the study populations varied from 70 to 16,848 women. Eight of the studies performed stratified analyses for nulli- and multiparity, and these studies included 8171 nulliparous and 20,581 multiparous women [1,17,18,20,21,23,25,26]. In 10 of the studies, indications for transfers were described [4,15-17,19-23,26].

All of the studies included women who had planned for, and were selected to have homebirth, at the onset of labour. Six of the studies were from settings where home births were an integrated and regulated part of the national or regional health care system [1,15,19,23,24,27], while the other studies described home births assisted by independent midwives. The studies from regulated settings described that only low-risk women were accepted for home birth, and some of the studies provided references to guidelines or other regulations [1,15,19,24]. In the independent settings, the proportion of women with high-risk pregnancies (e.g., post-term delivery, previous caesarean section, or medical conditions that may affect birth outcomes) varied from 4–17% in the four studies, with detailed descriptions of the study populations [4,18,25,26].

One of the studies was assessed as good quality [1], and the others as medium quality. Study characteristics and quality assessments are shown in Table 1.

**Table 1:** Description and quality assessments of included studies

**Study**	**Inclusion criteria**	**Participants (% P0**^**1**^**)**	**Caregivers**	**Study design**	**Data source**	**Duration of observation time after birth**	**Analyses stratified for parity**	**Study population representative**	**Quality**
Amelink-Verburg *et al.*[24]	All women under midwifery care and with an intended home birth in the Netherlands during 01.01.2001-31.12.2003	N = 168,618	Primary level midwifes	Prospective	The Dutch Midwifery Perinatal Database (LVR1)	2 h after the birth of the placenta	No	Data from LVR1 covers 95% of midwifery practices.	Medium
		(Parity not described)							
Anderson *et al.*[22]	All Nurse-midwifery practices in the USA during 1987-1991	N = 11,084	Independent midwives	Retrospective	Data collection forms from the midwives	..”early postpartum period”	No	66% of midwifery practices participated.	Medium
		(Parity not described)							
BECG^2^[1]	All NHS trusts providing intrapartum care at home in England (UK) during April 2008-April 2010	N = 16,840	National Health Service midwives	Prospective	Data collection forms from midwives and hospitals	48 h postpartum	Yes	97% of trusts providing home birth services participated. (Home births attended by independent midwives in the region were not included)	Good
		(27.2%)							
Blix *et al.*[25]	All planned home births in Norway during 01.01.1990-31.12.2007	N = 1631	Independent midwives	Retrospective	Midwives’ patient files	5 days postpartum	Yes	Unclear, probably >70% of all planned home births during the study period	Medium
		(22.6% )							
Davies *et al.*[23]	All women in the North Regional Health Authority area (UK) who planned for a home birth and expected to deliver in 1993	N = 177	National Health Service midwives	Prospective	Data collection forms from midwives, women and GP’s	Not described	Partly	Unclear, probably were all planned home births attended by NHS midwives included.	Medium
		(9.1%)							
Hansen and *et al.*[27]	All home births assisted by midwives employed by the local health authorities in the Municipality of Copenhagen (Denmark) during 1980-1982	N = 102	Midwives employed at Hvidovre Hospital	Retrospective	Hospital patient files	Not described	No	All planned home births assisted by midwives employed by the local health authorities were included. (Home births attended by independent midwives in the region were not included)	Medium
		(about 50%)							
Howe [17]	All home births attended by a registered midwife in the south-west of Western Australia during 01.01.1983-31.12.1986	N = 165	Independent midwives	Retrospective	Midwifery registers	Not described	Partly	All midwives participated	Medium
		(31.5%)							
Hutton *et al.*[18]	All home births attended by Ontario midwives during 01.04.2003-31.03.2006 (Canada)	N = 6,692	Certified midwives who are required to submit all data to a regional database	Retrospective	The Ontario Ministry of Health Database	Not described	Partly	All planned home births were included	Medium
		(34.3%)							
Johnson and Daviss [4]	All home births involving certified professional midwives across the USA and Canada during 01.01.2000-31.12.2000	N = 5,418	Independent midwives	Prospective	Data collection forms from the midwives	Not described	No	73% of the midwives asked, participated. <1% of the women declined participation	Medium
		(31.2%)							
Janssen *et al.*[19]	All planned home births attended by regulated midwives in British Columbia (Canada) during 01.01.1998-31.12.1999	N = 797	Regulated midwives	Prospective	Data collection forms	Not described	No	>99% of the data collection forms were received	Medium
		(about 47%)							
Lindgren *et al.*[26]	All planned home births in Sweden during 01.01.1992-31.07.2005	N = 1,025	Independent midwives	Retrospective	Data collection forms to the mothers	…”shortly after planned home birth”	Yes	99% of the women asked, agreed to participate. Unclear if all home births were identified.	Medium
		(23.8%)							
McMurtrie *et al.*[15]	The first 100 booked home births at the St. George Homebirth Program during Nov 2005-March 2009 in New South Wales (Australia)	n = 70 attempted home births	Midwives employed at St George Hospital	Prospective	Databases at the birth centre	Not described	No	All planned homebirths were included. (Home births attended by independent midwives in the region were not included)	Medium
		(Parity not described)							
Murphy *et al.*[21]	All nurse-midwifery practices providing home birth services in the USA during Dec 1994-Dec 1995	N = 1,221	Independent midwives	Prospective	Data collection forms from the midwives, data from hospital files	Not described	Partly	64% of midwifery practices participated. 20% of women transferred to hospital were lost-to-follow-up	Medium
		(22.0%)							
Parratt *et al.*[16]	All planned home births in Victoria (Australia) during 1995-1998	N = 419	Independent midwives	Retrospective	Midwives’ patient files	Not described	No	50-60 births were not included	Medium
		(about 31%)							
Tyson [20]	All planned midwife-attended home births in Toronto (Canada) during Jan 1983-Jul 1988	N = 1,001	Independent midwives	Retrospective	Midwives’ patient files	4 days postpartum	Yes	All midwives participated	Medium
		(Parity not described)							

^1^P0 = nulliparous women. ^2^Birthplace in England Collaborative Group.

### Heterogeneity, robustness, and risks for bias

We detected considerable heterogeneity across the studies through reading the studies, and inspecting tables and forest plots. *I*^2^ was above 90% in most of the outcomes (Additional file 3). The reason for this finding is probably because of differences in study populations and clinical practice (e.g., guidelines and traditions for transfer indications).

We performed sensitivity analyses by comparing the pooled prevalence when the largest study was excluded [24], in studies where parity was described [1,4,17-19,21,23,25-27], in studies where parity was not described [15,20,22,24], in studies where home births were booked with independent midwives [3,4,16-18,20-22,25], in studies from settings where home births were an integrated part of the regional or national health care system [1,15,19,23,24,27], and in studies with 10,000 to 100,000 women included [1,22], with 1000 to 10,000 women included [3,4,20,21,25], and with less than 1000 women included [15-17,19,23,27]. In some cases, there were considerable differences between the estimates when comparing a fixed-effect model and a random-effect model, which indicated large differences in results and study sizes. Estimation of the proportion of women transferred to hospital lacked robustness across the sensitivity analyses, while estimation of the proportion of women and neonates transferred for foetal distress, postpartum haemorrhage, respiratory problems, and emergency transfers remained more stable (Additional file 3).

Risks of selection bias are linked to what degree the study populations are representative for all planned home births in the country or region. Some of the studies did not include all home births in the country or region, and it is unclear if the study populations were representative for the total populations [4,16,21,22,25] (Table 1).

Prospective data collection usually provides better study quality than retrospective data collection. Seven of the 15 included studies had a prospective study design [1,4,15,19,21,23,24] (Table 1).

Because of heterogeneity and lack of robustness across the studies, there were considerable risks for bias if performing meta-analyses of the prevalence of transfers (Additional file 3). Therefore, we decided to descriptively present the findings.

### All transfers

The total proportion of women transferred to hospital during labour or after birth, varied from 9.9% to 31.9% across the studies (Table 2).

**Table 2:** Outcome events and prevalence of transfers from home to hospital in planned home births

	**Outcome events, (n/N)**	**Prevalence**	**(95% CI)**
**All transfers**^**1,2**^			
Amelink-Verburg *et al.*[24]	53809/168618	31.9	(31.7-32.1)
Anderson *et al.*[22]	1093/11081	9.9	(9.3-10.4)
BECG^3^[1]	3530/16840	21.0	(20.3-21.6)
Blix *et al.*[25]	197/1631	12.1	(10.5-13.8)
Davies *et al.*[23]	39/177	22.0	(16.2-28.9)
Hansen *et al.*[27]	29/102	28.4	(19.9-38.2)
Howe [17]	34/165	20.6	(14.7-27.6)
Hutton *et al.*[18]	954/6692	14.3	(13.4-15.1)
Janssen *et al.*[19]	165/797	20.7	(17.9-23.7)
Johnson and Daviss [4]	655/5418	12.1	(11.2-13.0)
Lindgren *et al.*[26]	128/1025	12.5	(10.5-14.7)
McMurtrie *et al.*[15]	10/70	14.3	(7.1-24.7)
Murphy *et al.*[21]	126/1221	10.3	(8.7-12.2)
Parratt *et al.*[16]	64/419	15.3	(12.0-19.1)
Tyson [20]	165/1001	16.5	(14.2-18.9)
**Transfers during labour**^**2**^			
Amelink-Verburg *et al.*[24]	40636/168618	24.1	(23.9-24.3)
Anderson *et al.*[22]	905/11081	8.2	(7.7-8.7)
BECG^3^[1]	2387/16840	14.2	(13.7-14.7)
Blix *et al.*[25]	156/1631	9.6	(8.2.11.1)
Davies *et al.*[23]	35/177	19.8	(14.2-26.4)
Howe [17]	23/165	13.9	(9.0-20.2)
Hutton *et al.*[18]	835/6692	12.5	(11.7-13.3)
Janssen *et al.*[19]	142/797	17.8	(15.2-20.7
Johnson and Daviss [4]	546/5418	10.1	(9.3-10.9)
Lindgren *et al.*[26]	109/1025	10.6	(8.8-12.7)
McMurtrie *et al.*[15]	7/70	10.0	(4.1-19.5)
Murphy *et al.*[21]	102/1221	8.4	(6.9-10.0)
Parratt *et al.*[16]	51/419	12.2	(9.2-15.7)
Tyson [20]	141/1001	14.1	(12.0-16.4)
**Transfers after birth**^**2**^			
Amelink-Verburg *et al.*[24]	3204/168618	1.9	(1.8-2.0)
Anderson *et al.*[22]	188/11081	1.7	(1.5-2.0)
BECG^3^[1]	1046/16040	6.2	(5.9-6.6)
Blix *et al.*[25]	41/1631	2.5	(1.8-3.4)
Davies *et al.*[23]	4/177	2.3	(0.6-5.7)
Howe [17]	12/165	7.3	(3.8-12.4)
Hutton *et al.*[18]	119/6692	1.8	(1.5-2.1)
Janssen *et al.*[19]	23/797	2.9	(1.8-4.3)
Johnson and Daviss [4]	37/5418	0.7	(0.5-0.9)
Lindgren *et al.*[26]	19/1025	1.9	(1.1-2.9)
McMurtrie *et al.*[15]	3/70	4.3	(0.9-12.0)
Murphy *et al.*[21]	24/1221	2.0	(1.3-2.9)
Parratt *et al.*[16]	13/419	3.1	(1.7-5.2)
Tyson [20]	24/1001	2.4	(1.5-3.5)
**Emergency transfers**			
Amelink-Verburg *et al.*[24]	5735/168618	3.4	(3.3-3.5)
Anderson *et al.*[22]	202/11081	1.8	(1.6-2.1)
Blix *et al.*[25]	16/1631	1.0	(0.6-1.6)
Davies *et al.*[23]	0/177	0.0	(0.0-2.1)
Hansen *et al.*[27]	1/102	1.0	(0.0-5.3)
Hutton *et al.*[18]	361/6692	5.4	(4.9-6.0)
Janssen *et al.*[19]	27/797	3.4	(2.2-4.9)
Johnson and Daviss [4]	185/5418	3.4	(2.9-3.9)
**Transfers for slow progress in labour**^**2**^			
Anderson *et al.*[22]	612/11081	5.5	(5.1-6.0)
Blix *et al.*[25]	108/1631	6.6	(5.5-7.9)
Anderson *et al.*[22]	13/177	7.3	(4.0-12.2)
Howe [17]	13/165	7.9	(4.3-13.1)
Janssen *et al.*[19]	56/797	7.0	(5.4-9.0)
Johnson and Daviss [4]	326/5418	6.0	(5.4-6.7)
Lindgren *et al.*[26]	66/1025	6.4	(5.0-8.1)
McMurtrie *et al.*[15]	6/70	8.6	(3.2-17.7)
Murphy *et al.*[21]	63/1221	5.2	(4.0-6.7)
Parratt *et al.*[16]	26/419	6.2	(4.1-9.0)
Tyson [20]	98/1001	9.8	(8.0-11.8)
**Transfers for fetal distress**^**2**^			
Anderson *et al.*[22]	170/11081	1.5	(1.3-1.8)
Davies *et al.*[23]	2/177	1.1	(0.1-4.0)
Howe [17]	2/165	1.2	(0.1-4.3)
Janssen *et al.*[19]	29/797	3.6	(2.5-5.2)
Johnson and Daviss [4]	119/5418	2.2	(1.8-2.6)
Lindgren *et al.*[26]	11/1025	1.1	(0.5-1.9)
McMurtrie *et al.*[15]	1/70	1.4	(0.0-7.7)
Murphy *et al.*[21]	13/1221	1.1	(0.6-1.8)
Tyson [20]	24/1001	2.4	(1.5-3.5)
**Transfers for PPH**^**2**^			
Anderson *et al.*[22]	44/11081	0.4	(0.3-0.5)
Davies *et al.*[23]	0/177	0.0	(0.0-0.2)
Howe [17]	1/165	0.6	(0.0-3.3)
Janssen *et al.*[19]	4/797	0.5	(0.1-1.3)
Johnson and Daviss [4]	34/5418	0.6	(0.4-0.9)
Lindgren *et al.*[26]	9/1025	0.9	(0.4-1.7)
McMurtrie *et al.*[15]	1/70	1.4	(0.0-7.7)
Murphy *et al.*[21]	3/1221	0.2	(0.0-0.7)
Parratt *et al.*[16]	6/419	1.4	(0.5-3.1)
Tyson [20]	7/1001	0.7	(0.3-1.4)
**Transfers for respiratory problems**^**2**^			
Anderson *et al.*[22]	62/11081	0.6	(0.4-0.7)
Howe [17]	1/165	0.6	(0.0-3.3)
Janssen *et al.*[19]	7/797	0.9	(0.4-1.8)
Johnson and Daviss [4]	33/5418	0.6	(0.4-0.9)
McMurtrie *et al.*[15]	1/70	1.4	(0.0-7.8)
Murphy *et al.*[21]	7/1221	0.6	(0.2-1.2)
Parratt *et al.*[16]	2/419	0.5	(0.1-1.7)
Tyson [20]	3/1001	0.3	(0.1-0.8)
**All transfers in settings where home births are an integrated and regulated part of the national or regional health care system**^**1,2**^			
Amelink-Verburg *et al.*[24]	53809/168618	31.9	(31.7-32.1)
BECG^3^[1]	3530/16840	21.0	(20.3-21.6)
Davies *et al.*[23]	39/177	22.0	(16.2-28.9)
Hansen *et al.*[27]	29/102	28.4	(19.9-38.2)
Janssen *et al.*[19]	165/797	20.7	(17.9-23.7)
McMurtrie *et al.*[15]	10/70	14.3	(7.1-24.7)
**All transfers in settings where the births were booked with independent midwives**^**1,2**^			
Anderson *et al.*[22]	1093/11081	9.9	(9.3-10.4)
Blix *et al.*[25]	197/1631	12.1	(10.5-13.8)
Howe [17]	34/165	20.6	(14.7-27.6)
Hutton *et al.*[18]	954/6692	14.3	(13.4-15.1)
Johnson and Daviss [4]	655/5418	12.1	(11.2-13.0)
Lindgren *et al.*[26]	128/1025	12.5	(10.5-14.7)
Murphy *et al.*[21]	126/1221	10.3	(8.7-12.2)
Parratt *et al.*[16]	64/419	15.3	(12.0-19.1)
Tyson [20]	165/1001	16.5	(14.2-18.9)
**Nulliparas, all transfers**^**1**^			
BECG^3^[1]	2057/4568	45.4	(44.0-46.9)
Blix *et al.*[25]	117/369	31.7	(27.0-36.7)
Howe [17]	14/52	26.9	(15.6-41.0)
Hutton *et al.*[18]	704/2293	30.7	(28.8-32.6)
Lindgren *et al.*[26]	57/244	23.4	(18.2-29.2)
Tyson [20]	116/360	32.2	(27.4-37.3)
**Nulliparas, transfers during labour**			
BECG^3^[1]	1605/4568	35.1	(33.8-36.5)
Blix *et al.*[25]	100/369	27.1	(22.6-31.9)
Davies *et al.*[23]	9/16	56.3	(29.9-80.2)
Hutton *et al.*[18]	638/2293	27.8	(26.0-29.7)
Lindgren *et al.*[26]	53/244	21.7	(16.7-27.4)
Murphy *et al.*[21]	73/269	27.1	(21.9-32.9)
Tyson [20]	102/360	28.3	(23,7-33.3)
**Nulliparas, transfers after birth**			
BECG^3^[1]	407/4568	8.9	(8.1-9.8)
Blix *et al.*[25]	17/369	4.6	(2.7-7.2)
Lindgren *et al.*[26]	4/244	1.6	(0.4-4.1)
Tyson [20]	14/360	3.9	(2.1-6.4)
**Multiparas, all transfers**^**1**^			
BECG^3^[1]	1472/12272	12.0	(11.4-12.6)
Blix *et al.*[25]	80/1262	6.3	(5.1-7.8)
Howe [17]	12/113	10.6	(5.6-17.8)
Hutton *et al.*[18]	250/4339	5.8	(5.1-6.5)
Lindgren *et al.*[26]	71/781	9.1	(7.2-11.3)
Tyson [20]	49/641	7.6	(5.7-10.0)
**Multiparas, transfers during labour**			
BECG^3^[1]	782/12272	6.4	(5.9-6.8)
Blix *et al.*[25]	56/1262	4.4	(3.4-5.7)
Davies *et al.*[23]	26/161	16.1	(10.8-22.8)
Hutton *et al.*[18]	197/4339	4.5	(3.9-5.2)
Lindgren *et al.*[26]	56/781	7.2	(5.5-9.2)
Murphy *et al.*[21]	54/952	5.7	(4.3-7.3)
Tyson [20]	39/641		
**Multiparas, transfers after birth**			
BECG^3^[1]	639/12272	5.2	(4.8-5.6)
Blix *et al.*[25]	24/1262	1.9	(1.2-2.8)
Lindgren *et al.*[26]	15/781	1.9	(1.1-3.1)
Tyson [20]	10/641	1.6	(0.8-2.9)

^1^“All transfers” refers to total transfers during labour and after birth.

^2^In both nulli- and multiparous women.

^3^BECG = Birthplace in England Collaborative Group.

In nulliparous women, the proportion of all transfers varied from 23.4% to 45.4%, and in multiparous women it ranged from 5.8% to 12.0%. There was a higher rate of transfer in studies from settings where home births were an integrated and regulated part of the national or regional health care system [1,15,19,23,24,27] than in settings with independent midwives [4,16-18,20-22,25,28] (Table 2).

### Transfer during labour

Most transfers to hospital occurred during labour and before the birth of the neonate. Across the 15 included studies, 8.2% to 24.1% were transferred. Seven studies that performed analyses stratified for nulli-and multiparity reported that 22.5% to 56.3% of all nulliparous women were transferred. In multiparous women, these proportions ranged from 4.4% to 16.1%.

Slow progress in labour was the most frequent indication for transfer in nulli- and multiparous women, occurring in 5.2% to 9.8% of all planned home births. Transfers because of foetal distress ranged from 1.0% to 3.6% (Table 2).

### Transfer after birth

Between 1.7% and 7.3% of women and neonates were transferred to hospital after birth. Four studies provided analyses stratified for parity; between 1.6% and 8.9% of nulliparous women and between 1.6% and 5.5% of the multiparous women were transferred after birth. Nine of the 15 included studies described the time span for transfers after birth, and this time varied from 2 hours to 5 days.

Between 0% and 0.2% of the women were transferred because of postpartum haemorrhage, and between 0.3% and 1.4% of neonates were transferred because of respiratory problems (Table 2).

### Emergency transfers

Eight of the included studies reported the proportion of emergency transfers, and it varied from 0% to 5.4% (Table 2).

The definitions of an emergency transfer varied across the studies. Some studies gave an overall definition, while others listed the indications defined as emergencies (Table 3).

**Table 3:** Definitions of “emergency transfer” across the studies

**Study**	**Study definitions of emergency transfers**
Amelink-Verburg *et al.*[24]	“…a referral for a complication that cannot be treated at the primary care level and that requires immediate diagnostics or treatment at the secondary care level” (Mother: Fetal distress, placental problems, abnormal presentation together with ruptured membranes, postpartum haemorrhage > 1000 ml, intrapartum fetal death. Neonate: early postnatal Apgar score >7 at 5 minutes, respiratory problems including meconium aspiration, congenital malformations with need of immediate care).
Anderson *et al.*[22]	Failure to progress, fetal distress, meconium in liquor, nonvertex presentations, postpartum haemorrhage, neonatal asphyxia, serious anomalies.
Blix *et al.*[25]	That the condition of the mother, fetus or infant demanded medical assistance as soon as possible.
Davies *et al.*[23]	Need for obstetric intervention within one hour after transfer.
Janssen *et al.*[19]	Fetal distress, meconium in liquor, breech presentation, active herpes, midwife not available, obstructed labour, retained placenta, repair episiotomy, postpartum haemorrhage, asphyxia, neonatal respiratory distress, distended abdomen in infant.
Johnson and Daviss [4]	Based on primary reason for transport.
Hansen *et al.*[27]	Poor fetal heart rate.
Hutton *et al.*[18]	Transported from home to hospital by ambulance during labour or immediately after delivery.

## Discussion

In the present review, we found that the proportion of transfer from home to hospital during and after planned home births varied from 9.9% to 31.9% across the study populations. In nulliparous, this proportion varied from 23.4% to 45.4%, and in multiparous, it ranged from 5.8% to 12.0%.

The proportion of transfer from home to hospital was higher in studies from settings where home births were an integrated part of the health care system compared with home births assisted by independent midwives. The study populations from regulated settings probably had slightly more nulliparous women included than in studies where independent midwives assisted births (Table 1). However, this was difficult to assess because not all of the studies reported parity. The proportion of nulliparous women in a study population affects transfer rates because nulliparous women are transferred more often than multiparous women. Another reason for the difference in transfer rates could be that in regulated settings, there are more strict guidelines for transfers and less room for individual assessments than in settings with independent midwives. In addition, in settings where home births are not part of the system, women might receive less information regarding home births. Those who choose home birth are probably a selected and motivated group, and less likely to be transferred. Assessment of what transfer rates should be to provide the best outcomes is difficult. Rates of transfer are not necessarily indicators of quality of care or a potential for adverse outcomes. High rates of transfer may be due to weather or traffic conditions, with the need for anticipatory planning. However, a low transfer rate may lead to cases of death and serious morbidity that could have been avoided. A high transfer rate may lead to unnecessary interventions and less patient satisfaction.

Whether there are different outcomes of home births in settings where home births are an integrated part of the health care system compared with home births assisted by independent midwives is unknown. A study from the UK compared outcomes of 1462 women assisted by independent midwives and 8676 women assisted by National Health Service midwives in all settings (obstetric units, midwifery-led units, and home births) [29]. Only 0.4% of the women assisted by National Health Service midwives gave birth at home, while 66.0% of the women assisted by independent midwives did so. These analyses did not adjust for place of birth, and the study design did not allow for conclusions in home births per se. This previous study found that although many outcomes were significantly better in women assisted by independent midwives compared with those assisted by National Health Service midwives, the perinatal mortality rate was higher among high-risk cases. When excluding high-risk cases from the analyses, there was no significant difference in the perinatal mortality rate between the two cohorts. The reasons for accepting high-risk cases in home birth settings should be further explored. This raises the issue of whether independent midwives are more willing to accept such women, or whether the women themselves are exerting pressure on midwives to accept them for home birth.

Our review showed that there was less variability across the included studies, and also less heterogeneity, when analysing transfers for specific indications, such as slow progress in labour, foetal distress, postpartum haemorrhage, and neonatal respiratory problems. One Canadian study reported a higher proportion of transfers because of foetal distress [19,20]. We could not find any methodological reasons why this study had a higher prevalence than the other studies.

Emergency transfers were differently defined across the studies. In one study, slow progress was one of the definitions for an emergency transfer [22]. However, this is usually not regarded as an emergency situation. In the study with the highest proportion of emergency transfers, the definition of an emergency transfer was if the mother or neonate was transported to hospital by ambulance [18]. To compare results across studies, having a standard definition of emergency transfers in planned home births would be useful. We considered that the definition of emergency transfer from the study in the Netherlands [24] was the best and most detailed (Table 3).

Women and neonates who experience emergency transfers during labour and immediately after the birth are probably a vulnerable group with higher risks for adverse outcomes. The studies in our review reported outcomes according to the principle of intention-to-treat, and provided no detailed description on outcomes in women and neonates after an emergency transfer. Mori et al. found that women who had planned for a home birth in England and Wales between 1994 and 2003, but were transferred to hospital, had the highest risk for intrapartum-related perinatal mortality. The authors emphasised that the results should be interpreted with caution because of inconsistencies in the recorded data [30]. A critical appraisal found weaknesses in the study design and that estimates of risk were inaccurate [31]. Evers et al. found an increased risk for perinatal death in women referred from midwifery care to obstetric care during labour in Utrecht in the Netherlands [32]. The results and conclusions of the study by Evers et al. [32] have also been discussed and questioned [33,34]. De Jonge et al. found that low-risk women with planned home births had a lower rate of severe maternal outcomes than those with planned hospital births [35]. Severe adverse outcomes were defined as postpartum haemorrhage >1000 ml, manual removal of the placenta and severe acute maternal morbidity (admission to an intensive care unit, eclampsia, blood transfusion of four or more packed cells, and other serious events). Among planned home births, severe acute maternal morbidity was 1.5/1000, postpartum haemorrhage occurred in 29.2/1000, and manual removal of the placenta occurred in 16.8/1000.

Performing audits to evaluate adverse outcomes during or after transfer to hospital would probably be useful. Audits may lead to improvements in health services (eg., better information between the home birth midwife and hospital, preventing delay in decisions, and transport plans).

Our study has a limitation. Four of the 15 included studies did not describe any indications for the transfers [1,18,24,27]. These four studies represented 89% of women included in the 15 studies.

## Conclusions

Future studies should report indications for transfer in planned home births, and also describe proportions and indications for emergency transfers. Analyses should be stratified for parity. Future studies also need to examine the difference in transfer rates in different settings.

## Abbreviations

n/N: Proportion; P0: Nulliparous; BECG: Birthplace in England collaborative group.

## Competing interests

The authors declare that they have no competing interests.

## Authors’ contributions

EB and HEL initiated and designed the study. EB performed the literature searches and all of the analyses. EB, MK, and HEL extracted data and assessed the literature. All of the authors participated in interpretation of results and participated in the writing process. EB revised the manuscript together with HEL, PØ, and MK. HK died in December 2013. All authors read and approved the final manuscript.

## Pre-publication history

The pre-publication history for this paper can be accessed here:

http://www.biomedcentral.com/1471-2393/14/179/prepub

## Supplementary Material

Additional file 1 Data extraction form. Click here for file

Additional file 2 Studies excluded after assessment in full text. Click here for file

Additional file 3 Sensitivity analyses. Click here for file
