# Supplementary material for: Transfer to hospital in planned home births: a systematic review
Source: BMC Pregnancy Childbirth. 2014 May 29;14:179. doi: 10.1186/1471-2393-14-179 (PMC4069085; doi:10.1186/1471-2393-14-179)
Supplement: Additional file 1 — Data extraction form. [file 1471-2393-14-179-S1.docx]

## Additional file 1: DATA EXTRACTION FORM

| Study: |  | | | |
| --- | --- | --- | --- | --- |
| Assessed by: |  | | | |
| Assessed date: |  | | | |
| When and where are data from? | |  | | Unclear |
| How were data collected? | |  | | Unclear |
| Lost-to-follow-up/did not participate, were not included | |  | | Unclear |
| Number of women planning homebirth | |  | | Unclear |
| Number (%) primiparas | |  | | Unclear |
| Number (%) multiparas | |  | | Unclear |
| Number (%) with known risk factors before onset of labour | |  | | Unclear |
| Number (%) with unplanned homebirth or ”freebirth” | |  | | Unclear |
| Describe the women panning for homebirth (risk status, sivil status, age, etc) | |  | | Unclear |
| What were the selection criteria for homebirths? | |  | | Unclear |
| Specify care in homebirths (midwife, physician, other, equipment, medicaments, etc) | |  | | Unclear |
| How many were selected to hospital during antenatal care (before onset of labour)? | |  | | Unclear |
| How were emergency transfers defined? | |  | | Unclear |
| RESULTS | | No. (%) | 95 % CI or comments etc | |
| Transfer to hospital after onset of labour and before the birth (all) | |  |  | |
| Indications | |  |  | |
| Transfer to hospital after onset of labour and before the birth (primiparas) | |  |  | |
| Indications | |  |  | |
| Transfer to hospital after onset of labour and before the birth (multiparas) | |  |  | |
| Indications | |  |  | |
| Transfer to hospital after the birth (all) | |  |  | |
| Indications | |  |  | |
| Transfer to hospital after the birth (primiparas) | |  |  | |
| Indications | |  |  | |
| Transfer to hospital after the birth (multparas) | |  |  | |
| Indications | |  |  | |
| Transfer to hospital after the birth, maternal indication (primiparas) | |  |  | |
| Indications | |  |  | |
| Transfer to hospital after the birth, maternal indication (multiparas) | |  |  | |
| Indications | |  |  | |
| Transfer to hospital after the birth, neonatal indication (primiparas) | |  |  | |
| Indications | |  |  | |
| Transfer to hospital after the birth, neonatal indication (multiparas) | |  |  | |
| Indications | |  |  | |
| Emergency transfers (all) | |  |  | |
| Indications | |  |  | |
| Emergency transfers (primiparas) | |  |  | |
| Indications | |  |  | |
| Emergency transfers (multiparas) | |  |  | |
| Indications | |  |  | |
| Describe emergency transfers, indications, etc | | | | |
| Were there other relevant outcome measures in the study? | | | | |
| Comments | | | | |
