# Supplementary material for: Transfer to hospital in planned home births: a systematic review
Source: BMC Pregnancy Childbirth. 2014 May 29;14:179. doi: 10.1186/1471-2393-14-179 (PMC4069085; doi:10.1186/1471-2393-14-179)
Supplement: Additional file 2 — Studies excluded after assessment in full text. [file 1471-2393-14-179-S2.docx]

| **Additional file 2 Studies excluded after assessment in full text** | | |
| --- | --- | --- |
| **Study** | | **Reason for exclusion** |
|  | Hinds MW, Bergeisen GH, Allen DT. **Neonatal outcome in planned vs unplanned out-of-hospital births in Kentucky.** *JAMA* 1985; **253:**1578-82. | Not relevant |
|  | Shearer JM. **Five year prospective survey of risk of booking for a home birth in Essex.** *Br Med J (Clin Res Ed)* 1985; **291:**1478-80. | Data from before 1980 |
|  | Hodnett ED, Abel SM. **Person-environment interaction as a determinant of labor length variables.** *Health Care Women Int* 1986; **7:**341-56. | No data on transfers |
|  | Schneider D. **Planned out-of-hospital births, New Jersey, 1978-1980.** *Soc Sci Med* 1986; **23:**1011-5. | Data from before 1980 |
|  | Schramm WF, Barnes DE, Bakewell JM. **Neonatal mortality in Missouri home births, 1978-84.** *Am J Public Health* 1987; **77:**930-5. | Data from before 1980, out-of-hospital births |
|  | Morse JM, Park C. **Home birth and hospital deliveries: a comparison of the perceived painfulness of parturition.** *Res Nurs Health* 1988; **11:**175-81. | Not relevant |
|  | Fleming AS, Ruble DN, Anderson V, Flett GL. **Place of childbirth influences feelings of satisfaction and control in first-time mothers.** *J Psychosom Obstet Gynaecol* 1988; **8:**1-17. | No data on transfers |
|  | Parazzini F, La Vecchia C. **Perinatal and infant mortality rates and place of birth in Italy, 1980.** *Am J Public Health* 1988; **78:**706-7. | Unplanned home births included |
|  | Abernathy TJ, Lentjes DM. **Planned and unplanned home births and hospital births in Calgary, Alberta, 1984-87.** *Public Health Rep* 1989; **104:**373-7. | No data on transfers |
|  | van Alten D, Eskes M, Treffers PE. **Midwifery in The Netherlands. The Wormerveer study; selection, mode of delivery, perinatal mortality and infant morbidity.** *BJOG* 1989; **96:**656-62. | Not relevant |
|  | Acheson LS, Harris SE, Zyzanski SJ. **Patient selection and outcomes for out-of-hospital births in one family practice.** *J Fam Pract* 1990; **31:**128-36. | Not relevant |
|  | Crotty M, Ramsay AT, Smart R, Chan A. **Planned homebirths in South Australia 1976-1987.** *Med J Aust* 1990; **153:**664-71. | Data from before 1980 |
|  | Woodcock HC, Read AW, Moore DJ, Stanley FJ, Bower C. **Planned homebirths in Western Australia 1981-1987: a descriptive study.** *Med J Aust* 1990; **153:**672-8. | Booked home births, not attempted |
|  | Anderson R, Greener D. **A descriptive analysis of home births attended by CNMs in two nurse-midwifery services.** *J Nurse Midwifery* 1991; **36:**95-103. | Booked home births, not attempted |
|  | Ford C, Iliffe S, Franklin O. **Outcome of planned home births in an inner city practice.** *BMJ* 1991; **303**:1517-9. | Data from before 1980 |
|  | Duran AM. **The safety of home birth: the farm study.** *Am J Public Health* 1992; **82:**450-3. | Data from before 1980 |
|  | Cunningham JD. **Experiences of Australian mothers who gave birth either at home, at a birth centre, or in hospital labour wards.** *Soc Sci Med* 1993; **36:**475-83. | No data on transfers |
|  | Woodcock HC, Read AW, Bower C, Stanley FJ, Moore DJ. **A matched cohort study of planned home and hospital births in Western Australia 1981-1987.** *Midwifery* 1994; **10:**125-35. | Booked home births, not attempted. Same cohort as Woodcock 1990 (double publication) |
|  | Janssen PA, Holt VL, Myers SJ. **Licensed midwife-attended, out-of-hospital births in Washington state: are they safe?** *Birth* 1994; **21:**141-8. | Not relevant |
|  | Declercq ER, Paine LL, Winter MR. **Home birth in the United States, 1989-1992. A longitudinal descriptive report of national birth certificate data.** *J Nurse Midwifery* 1995; **40:**474-82. | Unplanned home births and freebirths included. |
|  | Pop VJ, Wijnen HA, van Montfort M, Essed GG, de Geus CA, van Son MM et al. **Blues and depression during early puerperium: home versus hospital deliveries.** *BJOG* 1995; **102:**701-6. | No data on transfers. |
|  | Ackermann-Liebrich U, Voegeli T, Gunter-Witt K, Kunz I, Zullig M, Schindler C et al. **Home versus hospital deliveries: follow up study of matched pairs for procedures and outcome. Zurich Study Team.** *BMJ* 1996; **313:**1313-8. | Booked home births, not attempted. Not intention-to-treat-analyses |
|  | Chamberlain G, Wraight A, Crowley P. *Home births.* London: The Parthenon Publishing Group, 1996. | Booked home births, not attempted. |
|  | Northern Region Perinatal Mortality Survey Coordinating Group. *Collaborative survey of perinatal loss in planned and unplanned home births.* *BMJ* 1996; **313:**1306-9. | Booked home births, not attempted. |
|  | Ngenda N, Khoo SK. **Failed homebirths: reasons for transfer to hospital and maternal/neonatal outcome.** *Aust N Z J Obstet Gynaecol* 1996; **36:**275-8. | Outcomes in transfers, no data about the entire study population or if the births were planned at home |
|  | Aikins MP, Feinland JB. **Perineal outcomes in a home birth setting.** *Birth* 1998; **25:**226-34. | No data on transfers |
|  | Bastian H, Keirse MJ, Lancaster PA. **Perinatal death associated with planned home birth in Australia: population based study.** *BMJ* 1998; **317:**384-8. | No data on transfers |
|  | Pang JW, Heffelfinger JD, Huang GJ, Benedetti TJ, Weiss NS. **Outcomes of planned home births in Washington State: 1989-1996.** *Obstet Gynecol* 2002; **100:**253-9. | Unclear if the homebirths were planned |
|  | Schirm E, Tobi H, de Jong-van den Berg LT. **Low use of medication in home deliveries in the Netherlands.** *Int J Gynaecol Obstet* 2002; **79:**5-9. | No data on transfers |
|  | McKenna P, Matthews T. **Safety of home delivery compared with hospital delivery in The Eastern Region Health Authority in Ireland in the years 1999-2002.** *Ir Med J* 2003; **96:**198-200. | No data on transfers  ( Poor study quality). |
|  | Bilcliff A. **Outcomes for planned homebirths in Victoria in 2003.** *Australian Midwifery News* 2004; **4:**18. | Booked home births, not attempted. Poor study quality |
|  | Der Hulst LA, van Teijlingen ER, Bonsel GJ, Eskes M, Bleker OP. **Does a pregnant woman's intended place of birth influence her attitudes toward and occurrence of obstetric interventions?** *Birth* 2004; **31:**28-33. | Booked home births, not attempted |
|  | Janssen PA, Carty EA, Reime B. **Satisfaction with planned place of birth among midwifery clients in British Columbia.** *J Midwifery Womens Health* 2006; **51:**91-7. | Booked home births, not attempted. |
|  | Christiaens W, Gouwy A, Bracke P. **Does a referral from home to hospital affect satisfaction with childbirth? A cross-national comparison.** *BMC Health Serv Res* 2007; **7:**109. | Booked home births, not attempted |
|  | Fullerton JT, Navarro AM, Young SH. Outcomes of planned home birth: an integrative review. *J Midwifery Womens Health* 2007; 52:323-33. | Review article |
|  | Andalucian Agency for Health Technology Assessment. Planned home birth: **Parto a domicilio programado. Situacion actual en los paises desarrollados.** Seville: Andalusian Agency for Health Technology Assessment (AETSA), 2007. | Review report |
|  | Blix E, Øian P, Kumle M. **Utfall etter planlagte hjemmefødsler**. *Tidsskr Nor Lægeforen* 2008; **128:**2436-9. | Review article |
|  | Christiaens W, Verhaeghe M, Bracke P. **Childbirth expectations and experiences in Belgian and Dutch models of maternity care.** *J Reproductive Infant Psychol* 2008; **26**:309-22. | Both home births and hospital births were included |
|  | Lindgren HE, Rådestad IJ, Christensson K, Hildingsson IM. **Outcome of planned home births compared to hospital births in Sweden between 1992 and 2004. A population-based register study.** *Acta Obstet Gynecol Scand* 2008; **87:**1-9. | No data on transfers |
|  | Mori R, Dougherty M, Whittle M. **An estimation of intrapartum-related perinatal mortality rates for booked home births in England and Wales between 1994 and 2003.** *BJOG* 2008; **115:**554-9. | Review article |
|  | Nove A, Berrington A, Matthews Z. **Home births in the UK, 1955 to 2006.** *Popul trends* 2008; **133**(Autumn):20-7. | No data on transfers |
|  | de Jonge A, van der Goes BY, Ravelli AC, Amelink-Verburg MP, Mol BW, Nijhuis JG et al. **Perinatal mortality and morbidity in a nationwide cohort of 529 688 low-risk planned home and hospital births.** *BJOG* 2009 **116:**1177-84. | No data on transfers |
|  | Gyte G, Dodwell M, Newburn M, Sandall J, Macfarlane A, Bewley S. **Estimating intrapartum-related perinatal mortality rates for booked home births: when the 'best' available data are not good enough.** *BJOG* 2009; **116:**933-42. | Critical appraisal |
|  | Janssen PA, Saxell L, Page LA, Klein MC, Liston RM, Lee SK. **Outcomes of planned home birth with registered midwife versus planned hospital birth with midwife or physician.** *CMAJ* 2009; **181:**377-83. | No data on transfers |
|  | Symon A, Winter C, Inkster M, Donnan PT. **Outcomes for births booked under an independent midwife and births in NHS maternity units: matched comparison study.** BMJ 2009; **338:**b2060. | Not relevant |
|  | van WC, van d, V, Lagro-Janssen T. **Home births revisited: the continuing search for better evidence.** *BJOG* 2009; **116:**1149-50. | Commentary article |
|  | Kennare RM, Keirse MJ, Tucker GR, Chan AC. **Planned home and hospital births in South Australia, 1991-2006: differences in outcomes.** *Med J Aust* 2010; **192:**76-80. | Transfer data only in cases of perinatal death |
|  | Declercq E, MacDorman MF, Menacker F, Stotland N. **Characteristics of planned and unplanned home births in 19 States.** *Obstet Gynecol* 2010; **116:**93-9. | No data on transfers |
|  | Fontein Y. **The comparison of birth outcomes and birth experiences of low-risk women in different sized midwifery practices in the Netherlands.** *Women Birth* 2010; **23:**103-10. | Both home births and hospital births were included |
|  | MacDorman MF, Menacker F, Declercq E. **Trends and characteristics of home and other out-of-hospital births in the United States, 1990-2006.** *Natl Vital Stat Rep* 2010; **58:**1-14, 16. | No data on transfers |
|  | Malloy MH. **Infant outcomes of certified nurse midwife attended home births: United States 2000 to 2004.** *J Perinatol* 2010; **30:**622-7. | No data on transfers |
|  | Ravelli AC, Jager KJ, de Groot MH, Erwich JJ, Rijninks-van Driel GC, Tromp M et al. **Travel time from home to hospital and adverse perinatal outcomes in women at term in the Netherlands.** *BJOG* 2011; **118:**457-65. | Not relevant |
|  | Wax JR, Pinette MG, Cartin A, Blackstone J. **Maternal and newborn morbidity by birth facility among selected United States 2006 low-risk births.** *Am J Obstet Gynecol* 2010; **202:**152-5. | No data on transfers |
|  | Wax JR, Lucas FL, Lamont M, Pinette MG, Cartin A, Blackstone J. **Maternal and newborn outcomes in planned home birth vs planned hospital births: a metaanalysis.** Am J Obstet Gynecol 2010; **203:**243-48. | Review article |
|  | Wax JR, Pinette MG, Cartin A. **Home versus hospital birth--process and outcome.** *Obstet Gynecol Surv* 2010; **65:**132-40. | Review article  ( double publication) |
|  | Chang JJ, Macones GA. **Birth outcomes of planned home births in Missouri: a population-based study.** *Am J Perinatol* 2011; **28:**529-36. | No data on transfers |
|  | Dahlen H, Schmied V, Tracy SK, Jackson M, Cummings J, Priddis H. **Home birth and the National Australian Maternity Services Review: too hot to handle?** Women Birth 2011; **24:**148-155. | No data on transfers |
|  | Davis D, Baddock S, Pairman S, Hunter M, Benn C, Wilson D et al. **Planned Place of Birth in New Zealand: Does it Affect Mode of Birth and Intervention Rates Among Low-Risk Women?** *Birth* 2011; **38:**111-9. | No data on transfers |
|  | MacDorman MF, Declercq E, Menacker F. **Trends and characteristics of home births in the United States by race and ethnicity, 1990-2006.** *Birth* 2011; **38:**17-23. | No data on transfers |
|  | van der Kooy J, Poeran J, de Graaf JP, Birnie E, Denktass S, Steegers EA et al. **Planned home compared with planned hospital births in the Netherlands: intrapartum and early neonatal death in low-risk pregnancies.** *Obstet Gynecol* 2011; **118:**1037-46. | No data on transfers |
|  | Lindgren HE, Rådestad IJ, Hildingsson IM. **Transfer in planned home births in Sweden--effects on the experience of birth: a nationwide population-based study.** *Sex Reprod Healthc* 2011; **2:**101-5. | Double publication |
|  | MacDorman MF, Declercq E, Mathews TJ. **United States home births increase 20 percent from 2004 to 2008.** *Birth* 2011; **38:**185-90. | No data on transfers,  (double publication) |
|  | Brown A, Booth C, Hall H. **Emergencies during home births.** *Pract Midwife* 2012; **15:**11-3. | The article does not contain empirical data |
|  | Schroeder E, Petrou S, Patel N, Hollowell J, Puddicombe D, Redshaw M et al. **Cost effectiveness of alternative planned places of birth in woman at low risk of complications: evidence from the Birthplace in England national prospective cohort study.** *BMJ* 2012; **344:**e2292. | No data on transfers |
|  | Chervenak FA, McCullough LB, Brent RL, Levene MI, Arabin B. **Planned home birth: the professional responibility response.** *AJOG* 2013; **208:** 31-8. | Review article |
|  | Tura G, Fantahun M, Worku A. **The effect of health facility delivery on neonatal mortality: systematic review and meta-analysis.** *BMC Pregnancy and Childbirth* 2013; **13:**18. | Review article |
|  | Olsen O, Clausen JA. **Planned hospital birth versus planned home birth.** *Cochrane Database of Systematic Reviews* 2012, Issue 9. Art. No.: CD000352. DOI: 10.1002/14651858.CD000352.pub2. | Review article |
|  | de Jonge A, Mesman JA, Manniën J, Zwart JJ, van Dillen J, van Roosmalen J. **Severe adverse maternal outcomes among low risk women with planned home versus hospital births in the Netherlands: nationwide cohort study.** *BMJ* 2013;**346:**f3263 doi: 10.1136/bmj.f3263. | No data on transfers |
