# Supplementary material for: Transfer to hospital in planned home births: a systematic review
Source: BMC Pregnancy Childbirth. 2014 May 29;14:179. doi: 10.1186/1471-2393-14-179 (PMC4069085; doi:10.1186/1471-2393-14-179)
Supplement: Additional file 3 — Sensitivity analyses. [file 1471-2393-14-179-S3.docx]

| **Additional file 3 Sensitivity analyses** |  |  |  |  |  |
| --- | --- | --- | --- | --- | --- |
| **Outcomes** | **Studies included in analysis** | **N** | **Inconsistency (I^2^)**  **(95% CI)** | **Pooled prevalence**  **Fixed effect model**  **(95% CI)** | **Pooled prevalence**  **Random effect model**  **(95% CI)** |
| **All studies (15 studies)** |  |  |  |  |  |
| All transfers [1, 4, 15-27] | 15 | 215257 | 99.8 (99.8-99.8) | 27.9 (27.7-28.1) | 17.0 (11.9-22.9) |
| Transfers during labour [1, 4, 15-26] | 14 | 215155 | 99.7 (99.7-99.7) | 21.1 (20.9-21.3) | 13.0 (9.2-17.5) |
| Transfers after birth [1, 4, 15-26] | 14 | 215155 | 98.6 (98.4-98.7) | 2.1 (2.1-2.2) | 2.6 (1.8-3.7) |
| Transfers for fetal distress [4, 15, 17, 19-23, 26] | 9 | 20955 | 73.2 (37.6-84.7) | 1.8 (1.6-1.9) | 1.8 (1.4-2.4) |
| Transfers for PPH [4, 15-17, 19-23] | 9 | 21374 | 46.7 (0-72.7) | 0.5 (0.4-0.6) | 0.6 (0.4-0.8) |
| Transfers for respiratory problems [4, 15-17, 19-22] | 8 | 20172 | 0 (0-56.3) | 0.6 (0.5-0.7) | 0.6 (0.5-0.7) |
| Emergency transfers [4, 18, 19, 22-25, 27] | 8 | 194516 | 97.0 (96.1-97.7) | 3.3 (3.2-3.4) | 2.5 (1.8-3.4) |
| All transfers, nulliparas [1, 17, 18, 20, 25, 26] | 6 | 7786 | 97.4 (96.5-98.0) | 39.0 (37.9-40.0) | 32.0 (24.4-40.2) |
| Transfers during labour, nulliparas [1, 18, 20, 21, 23, 25, 26] | 7 | 8119 | 90.8 (83.9-93.9) | 31.7 (30.7-32.7) | 29.0 (24.8-33.4) |
| Transfers after birth, nulliparas [1, 20, 25, 26] | 4 | 5541 | 93.2 (86.1-95.9) | 7.8 (7.1-8.6) | 4.7 (1.9-8.6) |
| All transfers, multiparas [1, 17, 18, 20, 25, 26] | 6 | 19408 | 97.3 (96.3-97.9) | 9.8 (9.4-10.2) | 8.4 (5.6-11.7) |
| Transfers during labour, multiparas [1, 18, 20, 21, 23, 25, 26] | 7 | 20408 | 87.3 (75.2-92.1) | 5.9 (5.6-6.2) | 6.2 (5.1-7.5) |
| Transfers after birth, multiparas [1, 20, 25, 26] | 4 | 14956 | 96.1 (93.4-97.3) | 4.5 (4.2-4.8) | 2.6 (1.0-4.9) |
| **When the biggest study [24] was excluded (14 studies)** |  |  |  |  |  |
| All transfers [1, 4, 15-23, 25-27] | 14 | 46639 | 98.4 (98.1-98.6) | 15.1 (14.8-15.5) | 15.8 (13.0-18.9) |
| Transfers during labour [1, 4, 15-23, 25, 26] | 13 | 46537 | 96.3 (95.4-97.0) | 11.6 (11.3-11.9) | 12.1 (10.3-14.0) |
| Transfers after birth [1, 4, 15-23, 25, 26] | 13 | 46537 | 98.4 (98.1-98.6) | 3.0 (2.8-3.1) | 2.7 (1.6-4.2) |
| Emergency transfers [4, 18-20, 23, 25, 27] | 7 | 25898 | 97.2 (96.3-97.8) | 2.9 (2.7-3.1) | 2.2 (1.1-3.7) |
| **In studies where parity was described (11 studies)** |  |  |  |  |  |
| All transfers [1, 4, 16-19, 21, 23, 25-27] | 11 | 34487 | 97.7 (97.2-98.1) | 17.0 (16.6-17.4) | 16.4 (13.5-19.6) |
| Transfers during labour [1, 4, 16-19, 21, 23, 25, 26] | 10 | 34385 | 93.4 (90.5-95.1) | 12.7 (12.3-13.0) | 12.4 (10.7-14.1) |
| Transfers after birth [1, 4, 16-19, 21, 23, 25, 26] | 10 | 34385 | 98.6 (98.3-98.8) | 3.5 (3.3-3.7) | 2.8 (1.4-4.7) |
| Transfers for fetal distress [4, 17, 19, 21, 23, 26] | 6 | 8803 | 94.7 (91.5-96.3) | 2.2 (1.9-2.5) | 3.3 (1.6-5.6) |
| Transfers for PPH [4, 16, 17, 16, 21, 23, 26] | 7 | 9222 | 36.0 (0-72.0) | 0.6 (0.5-0.8) | 0.6 (0.4-0.9) |
| Transfers for respiratory problems [4, 16, 17, 19, 21] | 5 | 8020 | 0 (0-64.1) | 0.6 (0.5-0.8) | 0.6 (0.5-0.8) |
| Emergency transfers [4, 18, 19, 23, 25] | 5 | 17985 | 96.6 (95.0-97.6) | 3.8 (3.5-4.1) | 2.4 (1.1-4.2) |
| **In studies where parity was not described (4 studies)** |  |  |  |  |  |
| All transfers [15, 20, 22, 24] | 4 | 180770 | 99.9 (99.9-99.9) | 30.2 (30.0-30.4) | 17.7 (5.8-34.3) |
| Transfers during labour [15, 20, 22, 24] | 4 | 180770 | 99.9 (99.8-99.9) | 22.9 (22.7-23.1) | 19.9 (5.2-26.0) |
| Transfers after birth [15, 20, 22, 24] | 4 | 180770 | 49.0 (0-81.6) | 1.9 (1.8-2.0) | 1.9 (1.7-2.1) |
| Transfers for fetal distress [15, 20, 22] | 3 | 12152 | 50.0 (0-84.5) | 1.6 (1.4-1.8) | 1.8 (1.2-2.6) |
| Transfers for PPH [15, 20, 22] | 3 | 12152 | 46.1 (0-83.7) | 0.4 (0.3-0.6) | 0.5 (0.3-1.0) |
| Transfers for respiratory problems [15, 20, 22] | 3 | 12152 | 14.7 (0-76.6) | 0.6 (0.4-0.7) | 0.5 (0.4-0.8) |
| Emergency transfers [22] | 1 | 11081 | - | - | - |
| **In studies from settings where home births were an integrated and regulated part of the national or regional health care system (6 studies)** |  |  |  |  |  |
| All transfers [1, 15, 19, 23, 24, 27] | 6 | 186604 | 99,5 (99,4-99,6) | 30,8 (30,7-31,0) | 23,3 (17,3-29,9) |
| Transfers during labour [1, 15, 24, 19, 23] | 5 | 186502 | 99,6 (99,6-99,9) | 23,1 (22,9-23,3) | 17,5 (11,7-24,0) |
| Transfers after birth [1, 15, 24, 19, 23] | 5 | 186502 | 99,5 (99,4-99,6) | 2,2 (2,1-2,3) | 3,5 (1,3-6,6) |
| Transfers for fetal distress [1, 19, 23] | 3 | 1044 | 45,1 (0-83,5) | 3,1 (2,1-4,2) | 2,6 (1,2-4,6) |
| Transfers for PPH [1, 19, 23] | 3 | 1044 | 14,3 (0-76,5) | 0,5 (0,2-1,1) | 0,5 (0,1-1,2) |
| Transfers for respiratory problems [15, 19] | 2 | 867 | Not applicable | 1,0 (0,5-1,8) | 1,0 (0,5-1,8) |
| Emergency transfers [19, 23, 27] | 3 | 1076 | 85,3 (26,9-93,3) | 2,4 (1,6-3,4) | 1,4 (0,04-4,7) |
| All transfers, nulliparas [1] | 1 | 16840 | - | - | - |
| Transfers during labour, nulliparas [1] | 1 | 16840 | - | - | - |
| Transfers after birth, nulliparas [1] | 1 | 16840 | - | - | - |
| All transfers, multiparas [1] | 1 | 16840 | - | - | - |
| Transfers during labour, multiparas [1] | 1 | 16840 | - | - | - |
| Transfers after birth, multiparas [1] | 1 | 16840 | - | - | - |
| **In studies from settings where home births were assisted by independent midwives (9 studies)** |  |  |  |  |  |
| All transfers [4, 16-18, 20-22, 25, 26] | 9 | 28653 | 93.1 (89.6-95.0) | 11.9 (11.5-12.2) | 13.1 (11.5-14.9) |
| Transfers during labour [4, 16-18, 20-22, 25, 26] | 9 | 28653 | 92.9 (89.2-94.9) | 9.9 (9.6-10.3) | 10.8 (9.3-12.4) |
| Transfers after birth [4, 16-18, 20-22, 25, 26] | 9 | 28653 | 89.4 (82.4-92.8) | 1.6 (1.5-1.8) | 2.1 (1.5-2.7) |
| Transfers for fetal distress [4, 17, 20-22, 26] | 6 | 19911 | 71.5 (5.1-85.8) | 1.7 (1.5-1.9) | 1.7 (1.3-2.1) |
| Transfers for PPH [4, 16, 17, 20-22, 26] | 7 | 20330 | 58.7 (0-80.2) | 0.5 (0.4-0.6) | 0.6 (0.4-0.8) |
| Transfers for respiratory problems [4, 16, 17,20-22] | 6 | 19305 | 0 (0-61.0) | 0.6 (0.5-0.7) | 0.6 (0.5-0.7) |
| Emergency transfers [4, 18, 22, 25] | 4 | 24822 | 98.5 (98.0-98.8) | 2.9 (2.7-3.1) | 2.7 (1.2-4.7) |
| All transfers, nulliparas [17, 18, 20, 25, 26] | 5 | 3318 | 44.8 (0-78.3) | 30.4 (28.8-32.0) | 29.9 (27.2-32.7) |
| Transfers during labour, nulliparas [18, 20, 21, 25, 26] | 5 | 3535 | 33.3 (0-77.4) | 27.4 (25.8-28.9) | 27.0 (24.7-29.3) |
| Transfers after birth, nulliparas [20, 25, 26] | 3 | 973 | 53.9 (0-85.4) | 3.6 (2.6-4.9) | 3.5 (2.0-5.4) |
| All transfers, multiparas [17, 18, 20, 25, 26] | 5 | 7136 | 74.9 (6.5-87.9) | 6.5 (5.9-7.0) | 7.3 (5.9-8.9) |
| Transfers during labour, multiparas [18, 20, 21, 25, 26] | 5 | 7975 | 66.9 (0-85.2) | 5.0 (4.6-5.5) | 5.4 (4.5-6.4) |
| Transfers after birth, multiparas [20, 25, 26] | 3 | 2608 | 0 (0-72.9) | 1.9 (1.4-2.4) | 1.9 (1.4-2.4) |
| **In studies with more than 100 000 included (1 study) [24]** | 1 | - | - | - | - |
| **In studies with 10 000-100 000 included (2 studies)** |  |  |  |  |  |
| All transfers [22, 24] | 2 | 179699 | Not applicable | 30.3 (30.1-30.5) | 19.7 (3.4-45.0) |
| Transfers during labour [22, 24] | 2 | 179699 | Not applicable | 22.9 (22.7-23.1) | 15.3 (3.3-33.8) |
| Transfers after birth [22, 24] | 2 | 179699 | Not applicable | 1.9 (1.8-2.0) | 1.8 (1.7-2.0) |
| Transfers for fetal distress [22] | 1 | 11081 | - | - | - |
| Transfers for PPH [22] | 1 | 11081 | - | - | - |
| Transfers for respiratory problems [22] | 1 | 11081 | - | - | - |
| Emergency transfers [22] | 1 | 11081 | - | - | - |
| All transfers, nulliparas | No studies | - | - | - | - |
| Transfers during labour, nulliparas | No studies | - | - | - | - |
| Transfers after birth, nulliparas | No studies | - | - | - | - |
| All transfers, multiparas | No studies | - | - | - | - |
| Transfers during labour, multiparas | No studies | - | - | - | - |
| Transfers after birth, multiparas | No studies | - | - | - | - |
| **In studies with 1000-10 000 included (6 studies)** |  |  |  |  |  |
| All transfers [4, 18, 20, 21, 25, 26] | 6 | 16988 | 84.7 (64.4-91.2) | 13.1 (12.6-13.6) | 12.9 (11.5-14.4) |
| Transfers during labour [4, 18, 20, 21, 25, 26] | 6 | 16988 | 87.8 (74.5-92.6) | 11.1 (10.6-11.7) | 10.8 (9.4-12.3) |
| Transfers after birth [4, 18, 20, 21, 25, 26] | 6 | 16988 | 90.7 (82.6-94.1) | 1.5 (1.3-1.7) | 1.8 (1.2-2.6) |
| Transfers for fetal distress [4, 20, 21, 26] | 4 | 8665 | 77.8 (0-89.9) | 1.9 (1.6-2.2) | 1.8 (1.1-2.4) |
| Transfers for PPH [4, 20, 21, 26] | 4 | 8665 | 35.9 (0-78.1) | 0.6 (0.5-0.8) | 0.6 (0.4-0.9) |
| Transfers for respiratory problems [4, 20, 21] | 3 | 7640 | 0 (0-72.9) | 0.6 (0.4-0.8) | 0.6 (0.4-0.8) |
| Emergency transfers [4, 18, 25] | 3 | 13471 | 99.4 (99.3-99.5) | 2.4 (2.1-2.6) | 1.8 (0.4-6.2) |
| All transfers, nulliparas [18, 20, 25, 26] | 4 | 3266 | 57.2 (0-83.8) | 30.4 (28.9-32.0) | 30.0 (26.9-33.1) |
| Transfers during labour, nulliparas [18, 20, 25, 26] | 4 | 3266 | 33.3 (0-77.4) | 27.4 (25.9-28.9) | 27.0 (24.7-29.3) |
| Transfers after birth, nulliparas [20, 25, 26] | 3 | 973 | 53.9 (0-85.4) | 3.6 (2.6-4.9) | 3.5 (2.0-5.4) |
| All transfers, multiparas [18, 20, 25, 26] | 4 | 7023 | 76.8 (0-89.5) | 6.4 (5.8-7.0) | 7.0 (5.6-8.6) |
| Transfers during labour, multiparas [18, 20, 21, 25, 26] | 5 | 7975 | 66.9 (0-85.2) | 5.0 (4.6-5.5) | 5.4 (4.5-6.4) |
| Transfers after birth, multiparas [20, 25, 26] | 3 | 2684 | 0 (0-72.9) | 1.9 (1.4-2.4) | 1.9 (1.4-2.4) |
| **In studies with < 1000 (6 studies)** |  |  |  |  |  |
| All transfers [15-17, 19, 23, 27] | 6 | 1730 | 60.2 (0-81.7) | 19.7 (17.9-21.6) | 20.0 (16.7-23.5) |
| Transfers during labour [15-17, 19, 23] | 5 | 1628 | 63.5 (0-84.0) | 15.8 (14.1-17.6) | 15.3 (12.1-18.7) |
| Transfers after birth [15-17, 19, 23] | 5 | 1628 | 44.1 (0-78.1) | 3.4 (2.6-4.4) | 3.7 (2.4-5.2) |
| Transfers for fetal distress [15, 17, 19, 23] | 4 | 1209 | 61.3 (0-87.0) | 2.9 (2.0-4.0) | 2.3 (0.9-4.3) |
| Transfers for PPH [15-17, 19, 23] | 55 | 1628 | 34.9 (0-77.9) | 0.7 (0.4-1.2) | 0.7 (0,2-1.4) |
| Transfers for respiratory problems [15-17, 19] | 4 | 1451 | 0 (0-72.9) | 0.8 (0.4-1.4) | 0.8 (0.4-1.4) |
| Emergency transfers [19, 23, 27] | 3 | 1026 | 85.3 (26.9-93.3) | 2.4 (1.6-3.4) | 1.4 (0-04-4.7) |
| All transfers, nulliparas [17] | 1 | 165 | - | - | - |
| Transfers during labour, nulliparas [23] | 1 | 177 | - | - | - |
| Transfers after birth, nulliparas | No studies | - | - | - | - |
| All transfers, multiparas [17] | 1 | 165 | - | - | - |
| Transfers during labour, multiparas | No studies | - | - | - | - |
| Transfers after birth, multiparas | No studies | - | - | - | - |
